# Reply to “A Gluten Reduction Is the Patients’ Choice for a Dietary ‘Bottom Up’ Approach in IBS—A Comment on “A 5Ad Dietary Protocol for Functional Bowel Disorders” *Nutrients* 2019, *11*, 1938”

**DOI:** 10.3390/nu12010140

**Published:** 2020-01-03

**Authors:** Fandi Ibrahim, Philippa Stribling

**Affiliations:** Life Sciences Directorate, School of Engineering, Arts, Science, and Technology, University of Suffolk, Ipswich IP4 1QJ, Suffolk, UK; p.stribling@uos.ac.uk

## 1. Introduction

We are grateful to Shaw et al. (2019) [1] for taking the time, effort, and interest to read our article; for highlighting the need for further research; and for initiating scientific debate regarding the dietary therapies currently used for functional bowel disorders (FBDs). We also agree with their view that streamlined approaches for tackling FBDs are warranted, which has been our raison d’être for developing a new simplified approach, the 5Ad Dietary Protocol (5AdD). In response to the authors’ valuable opinion, we will address their raised concerns, delineate any further concerns that might arise from other experts, and give our insight to encourage further research in this important arena of human health.

## 2. A Bottom-Up or Top-Down Approach?

We feel that debating the concept of a bottom-up or top-down approach focuses heavily on semantic aspects, causing the scientific merit and fundamental dietary aspects of the 5AdD to be overlooked, perhaps unnecessarily, in our humble view. However, we wish to clarify that a bottom-up approach was adopted in selecting more than 57 raw food items based on their lack of association with FBDs, as evidenced from the available literature in many instances, and on a theoretical basis (e.g., pre-post agriculture approach), with the purpose of forming a complementary diet for long-term use for people with FBDs. For instance, considering that root tubers have been a part of the human diet for much longer than cereals and legumes, the first were chosen in preference over the latter for inclusion within the 5AdD [2,3,4,5,6].

## 3. How to Develop a Dietary Therapy for FBDs

Firstly, we need to portray our view that FBDs are a spectrum of food intolerances caused by intrinsic (genetic or post-disease) factors and extrinsic dietary factors [7,8,9]. Unfortunately, all current dietary therapies originate from a symptom-based approach owing to the lack of reliable diagnostic biomarkers, apart from lactose intolerance and some rare genetic diseases (e.g., sucrose-isomaltase deficiency) [9,10,11,12]. Even in these examples, positive test results do not necessarily match with symptom severity [13]. It is worth mentioning that the nutrigenetics/nutrigenomics approach is still far from being applied in this area owing to the complex gene–environment interactions, and immunoglobulin testing is also unreliable [14,15]. Therefore, a symptom-relief-based approach is currently the cornerstone in developing dietary therapies for FBDs. This is not a perfect solution, as the absence of symptoms does not preclude any negative long-term effects (e.g., chronic low-grade inflammation and/or enhanced gut permeability), and more research should focus on finding reliable and diet-responsive biomarkers in this area.

Thus, the 5AdD was developed by theorising that the majority of FBDs are likely to be a form of food intolerance owing to the introduction of relatively new foods to the human diet (post-agricultural era). This view is perhaps beneficial to the patients’ perception of their symptoms, and it may be more realistic and acceptable than the newly suggested term by the Rome IV consensus of “disorders of gut–brain interaction” [10], which is definitely more stigmatising than “functional” or “intolerance”, contrary to the intended purpose. An example to further clarify this approach is the fact that the minority of the world’s population who are tolerant to lactose are actually those who developed a beneficial mutation, whereas 75% of the world’s population with primary lactose intolerance have the normal genotype [11,16]. Hence, it is not biologically inconceivable that the high prevalence of FBDs is a direct result of intolerances to newly introduced foods, particularly with the ubiquities consumption of grains and pulses in our modern diet. In favour of the “food intolerance to the modern diet” concept, it is worth noting that 10% of Greenland Eskimos and 0.2% of North Americans have congenital sucrase-isomaltase deficiency, which usually ends up being diagnosed as irritable bowel syndrome (IBS) [17]. During the 5AdD development, we have been working on the assumption that food intolerances cannot be cured, at least currently, and that dietary exclusion is likely the most beneficial approach.

## 4. The Similarities Between the 5AdD and Current Dietary Therapies

The 5AdD comprises multiple built-in dietary therapies for FBDs (e.g., the low FODMAP diet (LFD), the gluten-free diet (GFD), and the low food chemical diet). Therefore, we hypothesised that the limited benefits evidenced from the current literature regarding these dietary therapies would probably be reinforced when combined [12]. The foods included within the 5AdD are not a random selection of food items, chosen by trial and error, but have instead been chosen based on the current evidence of the available dietary therapies, as well as some novel aspects unique to the 5AdD. These factors, combined, are likely responsible for the significant improvements observed within a week in the studied group [18]. Thus, the 5AdD is deemed a natural development, built on the common dietary therapies, and aims to streamline the delivery and adherence without compromising safety and nutritional adequacy [18]. 

## 5. The Differences between the 5AdD and Current Dietary Therapies

The 5AdD has the following distinct features compared with the LFD and GFD:

1. Compositional features: all-natural foods, 1 kg of fruit and vegetables per day, nuts and seeds, animal protein from terrestrial and marine sources, fermented foods, low salt, high K/Na ratio, no refined oil, no refined carbohydrate, nearly zero added/free sugar, zero artificial trans-fat, minimally processed foods, and healthy cooking (boiling and steaming).
2. The duration of the intervention phase is only 1–2 weeks; we have seen success within a week and there is ongoing research involving a two-week intervention period to ascertain the benefits seen in the first study.
3. If the patient observes a significant improvement, the intervention phase can be pursued indefinitely at the patient’s choice—we do not insist, encourage, nor discourage a re-challenge, as it is very unlikely for a person who suffers from FODMAP sensitivity, for instance, to ever be healed/cured, despite some potential adaptation. However, we have still given the individual the opportunity of trying to optimise their food selection, guided by post-intervention instructions. The lack of concern in following the 5AdD in the long-term lies in its approach of complementary foods within the five groups, assuming the inclusivity within each group as instructed.
4. In addition to FODMAPs and gluten, the 5AdD pays attention to some other dietary intolerance aspects that are not commonly considered; as such, in the intervention phase, we removed foods rich in resistant proteins (e.g., α-amylase inhibitors, trypsin inhibitors, and dietary lectins) and other proteins from the prolamin superfamily, as well as food additives (e.g., carboxymethyl cellulose, xanthan gum, and carrageenan) [9,19,20]. To enable this, the diet was designed to be majorly free from any processed or refined foods and, additionally, all cereals and pulses were excluded. Cold-pressed oils were not considered refined foods.
5. Coffee is excluded (in both decaffeinated or caffeinated form) owing to, at least, its stimulatory effects on rectosigmoid motor activity [21,22].
6. Low-lactose dairy products are included in the 5AdD to improve nutritional adequacy, considering the common tolerability of low lactose intake (up to 20 g per day) among lactose intolerant individuals [8,11,13].
7. The 5AdD could easily be delivered by a nutritionist, providing they have access to any nutritional analysis software, rather than solely by a dietitian.

## 6. Is the LFD Alone the Ultimate Choice for People with FBDs?

As we believe that FBDs are a spectrum of multifactorial food intolerances, it makes sense that a large number of FBD sufferers would benefit from the LFD, as it excludes a wide range of offending food components (i.e., FODMAPs). However, as FODMAPs are all within the carbohydrate category, there are still people who would not benefit from a LFD approach if, for example, their symptoms arise in response to gluten, dietary lectins, or food additives/chemicals. Additionally, the requirement of supervision from a dietitian for those following the LFD implies inherent safety concerns with the LFD and also confirms our own and the authors’ published view about the LFD [18], that is, Shaw et al. (2019) [1]. A recent meta-analysis concluded that very low-quality evidence exists that the LFD is effective in reducing symptoms in IBS patients [23]. 

There is a large population of IBS patients that follow dietary manipulation from advertising, lay press, and the internet [12]. As most of the information that is easily accessible to the public regarding the LFD is a long list of foods that cannot be consumed (the content of which is often conflicting between articles), it is not unreasonable to suggest that nutritional inadequacies (e.g., calcium) can occur following a self-led LFD [24,25]. Without a healthcare professional or dietitian that can ensure proper nutrition throughout the three phases, it is likely that nutritional inadequacies can occur, especially as only 40% of patients have been shown to follow the LFD correctly [26]. Additionally, because the restrictive phase of the LFD is usually implemented for around 4–8 weeks, and because of the complicated nature of the diet (especially when self-administered), there is a high risk of low adherence [27,28,29].

## 7. Is the GFD Alone the Ultimate Choice for People with FBDs?

There is a subgroup of FBD sufferers whose food intolerances are gluten-related and would, therefore, benefit from a GFD, but these individuals would make up a small percentage of FBD sufferers considering the masked role of fructan in wheat products [30,31]. It is, therefore, unsurprising that there is insufficient evidence to support claims that the GFD reduces IBS symptoms, and it is clear that the GFD is only appropriate for a subgroup of those with IBS [23,32]. This leads us to disagree with the authors’ statement that the GFD is beneficial for all people with FBDs. 

The GFD cannot be a biologically plausible solution alone, as it is in direct contradiction with the aetiological role played by FODMAPs in symptom generation. A low-fructan and low-gluten diet cannot come under an umbrella term of the GFD, as the authors insinuate, as fructans exist in various food items (e.g., garlic, onion, and artichoke), not only in wheat products [33]. The non-wheat fructan-containing foods are naturally gluten-free, but still high in FODMAPs, and would not be excluded on a GFD. Additionally, the GFD excludes only a few types of cereals and includes all the legume family; legumes are rich sources of oligosaccharides (e.g., raffinose, verbascose, and stachyose), which are probably the most potent FODMAPs for those with FBDs [34]. The authors assume that reducing gluten will always reduce fructans, but this is not the case, as discussed above. They also appear to be contradicting themselves as they affirm the benefits of the LFD, but do not consider FODMAP avoidance within the GFD.

Furthermore, the majority of individuals following a GFD replace gluten-containing foods with specialised gluten-free products that may well contain resistant proteins or phylogenetically similar proteins to gluten, instead of changing their diet to only include naturally gluten-free products. For instance, rice, maize, and oats contain prolamins from the same prolamin superfamily that contains gluten and closely-related proteins [35], which might explain the lack of response to the GFD for some individuals. Interestingly, the concerns about the nutritional adequacy of the GFD were raised recently by some authors of the Comment [36]. Also, constituents of gluten-free cereal (rice and corn) have lower levels of dietary fibre, protein, and folate [37,38]. Furthermore, gluten-free products are normally more expensive than their gluten-containing counterparts [39]. 

## 8. Is the 5AdD Restrictive or a Corrective Action?

The assessment of food restriction and variety should always be considered in terms of the distinct raw food materials and how they collectively form a diet that meets our nutritional requirements. To put it simply, a person could be consuming over 100 different food items per week, but 70% of these food items may be cereal-based (e.g., bread, rice, and pasta), which is still a restrictive diet, and they may still be deficient in some essential nutrients, despite eating what appears to be a varied diet. On the other hand, another person could consume a handful of carefully selected distinct raw food items (e.g., by following the 5AdD) but, instead, meet all of their essential nutrient requirements. However, there is no scientific evidence or consensus to advocate a specific number of foods that should be included in the diet, and the concept of variety is carefully built into the design of the 5AdD. When judging the quality of eating using a universal scoring system, such as the Healthy Eating Index (HEI) and nutrient profiling schemes, only the composition/nutrient density of foods is considered, instead of a single specific food or the total number of foods [40,41]. 

The 5AdD can hardly be viewed as a restrictive approach when applying the above-mentioned objective measures, and because it contains more than 57 distinct raw foods (nutrient-dense) including meats, eggs, dairy, white and oily fish, seafoods, tubers, nuts, seeds, vegetables, fruits, and natural flavourings and drinks. Indeed, evidence from the food frequency questionnaire (FFQ) data, collected from our study group at baseline, showed that the total number of different food items consumed ranged from only 9 to 48 items, with a median of 23.5 items over a year when counting the frequency of all foods consumed at least twice a week. These food items included sugar, honey, crisps, juices, and various nutrient-poor foods. This, again, shows that the 5AdD cannot really be viewed as a restrictive diet, and the concerns regarding those following the 5AdD are unwarranted.

The 5AdD could be viewed rather as a corrective action, nutritionally speaking, in addition to being an approach that provides gut symptom relief. It is hardly conceivable that a diet with the compositional features mentioned earlier (Section 5) would be a matter of concern. These features are the core of all dietary guidelines worldwide, and the difficulty lies only in the adherence to the 5AdD, rather than any concerns or risks associated with it. In fact, the U.K. National Diet and Nutrition Survey (NDNS) showed that the general population, on average, have been consistently eating lower than the recommended intake of fruit and vegetables, oily fish, and fibre, and there was a downward trend in intake of most vitamins and minerals between 2008/09 and 2016/17, while on the other hand, the mean salt and saturated fatty acids intake was consistently higher than the target intake [42]. The consumption of red meat remained above the recommended maximum of 70 g per day for adult men, and sugar intake remained at least double the maximum recommendation over the same periods mentioned above [42,43]. The HEI showed that the overall score is only 59 of 100 when judged by the healthy eating guidelines for Americans [44]. Thus, a carefully planned diet, such as the 5AdD, should be of no concern.

The “model” diet referred to by Shaw et al. (2019) [1] was an actual experiment whereby a participant religiously followed a prescribed strict diet, based on the 5AdD. Although we are unable to provide evidence of long-term adherence yet, we do expect deviation from the 5AdD model diet and understand that the adherence level is an essential part of the success of any dietary therapies. The 5AdD, like any other dietary therapies, will face challenges such as behavioural and cultural changes in eating habits, as it is not commensurate with ready meals, fast foods, refined foods, and the commonly consumed restaurant foods. To eat all-natural foods nowadays is a big challenge, and we are working to improve this by adding behavioural change elements and enhanced meal sensory properties (for instance, we have added 15 herbs and spices to the protocol).

## 9. To Exclude or Not to Exclude Food Groups?

Excluding a food group has always been a controversial subject when it comes to any dietary therapy, while in fact, what matters is not the food group per se, but the delivery of the essential nutrients, in their modern concept, at an optimal level (e.g., not only the known essential nutrients that the body cannot synthesise, but also dietary fibre and various phytochemicals) [45]. Excluding cereals and legumes may seem as if there would be a reduction in plant foods in the diet, but careful examination of the 5AdD would show that the recommendation of consuming 1 kg of fruit and vegetables per day alone, and adding the nuts and seeds and root tuber groups, should defuse any concerns in regard to fibre intake and the relative contribution of plant to animal foods. Carefully planned eating can overcome any shortcomings of missing a food group, and that was inherent in the design of the 5AdD. For instance, people may be vegetarian, or even vegan, and can still meet all nutritional requirements with careful planning and supplementation, while the basis of the 5AdD is much less concerning. There is no evidence for any harm caused from the exclusion of food groups, providing there is an alternative and nutritional requirements are met. The reason for excluding food groups during the 5AdD is because of their FODMAP and resistant protein (e.g., dietary lectins) content, in addition to the theoretical basis of their relatively recent inclusion in human diet [4,5,9,12,20]. 

## 10. Resistant Proteins—Beyond Gluten

As detailed above, one of the novel aspects of the 5AdD is its focus on resistant proteins as a whole, rather than focusing only on gluten, which may explain, in part, the lack of improvement in non-responders to the current therapies. Resistant proteins are commonly found in cereals and legumes and include prolamins, lectins, and α-amylase inhibitors (ATIs). These are resistant to proteolysis in the small intestine [46,47] and their digestion results in pathogenic peptide fragments in susceptible individuals. Generally speaking, these are known to induce the release of pro-inflammatory cytokines, and cause damage to the intestinal epithelial layer [25]. ATIs represent 2%–4% of total wheat protein and are believed to induce an innate immune response via activation of the Toll-like receptor 4 (TLR4) on immune cells within the intestine. They have also been shown to be involved in an adaptive immune response, have been implicated in coeliac disease, and may also contribute to inflammation in other disorders [48,49,50,51]. Additionally, legumes, which have a higher protein content than cereals, contain significant levels of anti-nutritional factors such as lectins and trypsin inhibitors, and two major storage proteins, vicilins and legumins, which are all resistant to proteolysis in the small intestine [46,52,53]. Legume lectins are said to be the most abundant group of the lectin family proteins and, like cereal lectins, are believed to cause damage to the intestinal epithelial layer, thereby affecting the absorption/utilisation of nutrients [52,53].

## 11. Is There a Role of Food Processing in FBDs? 

Both cereal and legume groups are likely to be involved in food intolerances in predisposed individuals, owing to their sensitivity/intolerance to gluten, FODMAPs, and/or resistant proteins. However, we encourage innovative ways of food processing, to mitigate these factors, and to make these foods more tolerable to those with FBDs. This would be viewed as a significant expansion of the ‘Free-From’ category, directed to the people with FBDs at an affordable cost. Food processing, such as soaking, germination, and sprouting, may potentially play an important role in tackling some challenges in this area [54,55].

## 12. Concluding Remarks

We believe that neither the GFD nor LFD are sufficient in terms of their long-term efficacy and nutritional adequacy, nor are they simplified enough to be applied without dietetic guidance. Therefore, we see an offering and a gap for the 5Ad Dietary Protocol to fill, employing its features of focusing on all-natural and nutrient-dense foods.

The 5Ad Dietary Protocol is the first dietary therapy to pay full attention to the potential role of resistant proteins in the aetiology of FBDs. This, and the combination of existing dietary therapies, is likely to be responsible for the promising results seen so far involving the 5Ad Dietary Protocol, but further research is needed in this area.

Finally, as the current dietary therapies for FBDs are symptom-relief-based, we welcome collaboration from other researchers to conduct more in-depth investigations focusing on the identification of diet-responsive biological markers for food intolerances.
